# Severe tyrosine-kinase inhibitor induced liver injury in metastatic renal cell carcinoma patients: two case reports assessed for causality using the updated RUCAM and review of the literature

**DOI:** 10.1186/s12876-022-02121-3

**Published:** 2022-02-05

**Authors:** Hana Studentova, Jindriska Volakova, Martina Spisarova, Anezka Zemankova, Kvetoslava Aiglova, Tomas Szotkowski, Bohuslav Melichar

**Affiliations:** 1 Department of Oncology, Palacký University Medical School and Teaching Hospital, Olomouc, Czech Republic; 2 Deparment of Clinical Pharmacology, Palacký University Medical School and Teaching Hospital, Olomouc, Czech Republic; 3 Second Department of Internal Medicine, Palacký University Medical School and Teaching Hospital, Olomouc, Czech Republic; 4 Department of Hemato-Oncology, Palacký University Medical School and Teaching Hospital, Olomouc, Czech Republic; 5 Institute of Molecular and Translational Medicine, Palacký University Medical School and Teaching Hospital, Olomouc, Czech Republic

**Keywords:** Renal cell carcinoma, TKI, Drug induced liver injury (DILI), Roussel Uclaf causality assessment method (RUCAM), Fatal liver failure, Clarithromycin, Hepatotoxicity

## Abstract

**Background:**

Sunitinib and pazopanib are both oral small molecule multityrosine kinase inhibitors (MTKI) used in the treatment of renal cell carcinoma (RCC). Hepatotoxicity or “liver injury” is the most important adverse effect of pazopanib administration, but little is known about the underlying mechanism. Liver injury may also occur in patients treated with sunitinib, but severe toxicity is extremely rare. Herein we report two new cases of severe liver injury induced by MTKI. Both cases are unique and exceptional. We assessed both cases for drug-induced liver injury (DILI) using the updated score Roussel Uclaf causality assessment method (RUCAM). The literature on potential pathogenic mechanisms and precautionary measures is reviewed.

**Case presentation:**

A case of a metastatic RCC (mRCC) patient treated with pazopanib who had manifestation of severe liver injury is presented. These manifestations consisted of grade 4 alanine aminotransferase (ALT) increase and grade 4 hyperbilirubinemia. Alternate causes of acute or chronic liver disease were excluded. The patient gradually recovered from the liver injury and refused any further therapy for mRCC. The patient was diagnosed with acute myeloid leukemia (AML) two years later and eventually succumbed to the disease. The second case describes a mRCC patient treated with sunitinib for 3,5 years and fatal liver failure after 2 weeks of clarithromycin co-medication for acute bronchitis.

**Conclusions:**

Liver injury has been commonly observed in TKI-treated patients with unpredictable course. Management requires regular routine liver enzyme-monitoring and the collaboration of medical oncologist and hepatologist. There is an unmet medical need for a risk stratification and definition of predictive biomarkers to identify potential genetic polymorphisms or other factors associated with TKI-induced liver injury. Any potential unrecommended concomitant therapy has to be avoided.

## Background

Since 2005, a number of targeted agents have been approved for the treatment of mRCC. Targeted drugs used in the first line setting include sunitinib, bevacizumab and pazopanib [1–3]. More recently, combination regimens based on immune checkpoint inhibitors were added to the front-line therapy of mRCC [4–8].

Pazopanib, an oral small molecule TKI targeting vascular endothelial growth factor receptors (VEGFR) 1, 2, and 3, stem cell factor receptor (SCF, c-kit), and platelet-derived growth factor receptors (PDGFR) α and β, is approved for the treatment of mRCC patients based on phase III trial that showed a significant prolongation in progression-free survival (PFS) compared to placebo in patients with advanced RCC [3]. A randomized phase III study comparing pazopanib with sunitinib in the first line setting of advanced RCC demonstrated similar treatment outcomes [9].

Liver injury is the most prominent adverse effect of pazopanib administration, but little is known about the underlying mechanism(s). Liver injury is a feared adverse event presenting as an isolated increase of serum transaminase activity, total bilirubin, or both in patients treated with pazopanib. In the registration trial, the incidence of any grade ALT elevation was 53%, with 12% incidence of grade 3 or 4 ALT elevation [3]. A subsequent meta-analysis of nine prospective trials of pazopanib showed incidence of peak ALT activity at ˃ 3 × upper limit of normal (ULN), ˃ 3-5xULN, ˃ 5-8xULN, ˃ 8-20xULN, and ˃20xULN in 20%, 8%, 5%, 5% and 1% of the patients, respectively [10]. Moreover, cases of fatal liver injury have also been reported [11].

Sunitinib is an oral small molecular weight inhibitor of VEGFR 1, 2, and 3, PDGFR α and β, KIT, Fms-like tyrosine kinase-3 (FLT3), colony stimulating factor receptor (CSF-1R), and the glial cell-line derived neurotrophic factor receptor (RET) approved for the treatment of advanced RCC in patients in the first line or second line setting [12]. Sunitinib is also approved for unresectable and/or metastatic gastrointestinal stromal tumor (GIST) after failure of imatinib treatment [13], and unresectable and/or metastatic well-differentiated pancreatic neuroendocrine tumors (pNET) [14]. Sunitinib is commonly administered orally once daily (OD) for 4 consecutive weeks, followed by a 2-week rest period, the recommended dose is 50 mg (schedule 4/2). Alternate regimen of sunitinib administration has also shown promising efficacy and a better safety profile. Dosing schedule 2/1 (2 weeks on/1 week off) has improved tolerability compared with the standard regimen (4/2) [15]. On the other hand, in pancreatic neuroendocrine tumors, sunitinib is taken orally OD without any rest period, and the recommended dose of sunitinib is 37.5 mg [13, 14].

Liver tests (LTs) abnormalities represent a relatively common event during the treatment with sunitinib. A meta-analysis in 5658 mRCC patients treated with sunitinib reported elevated liver enzymes in 40% of the patients, while grade 3 and 4 liver injury occurred in 3% [16]. Fulminant liver failure associated with sunitinib treatment is rare. Several cases of serious sunitinib-induced acute liver failure, including fatal cases, have been reported in the literature [17–22]. A meta-analysis of 3691 patients focusing on the incidence and relative risk of liver injury in patients treated with anti-angiogenic TKIs found that liver injury of sunitinib, pazopanib or other anti-angiogenic TKIs did not depend on the type of disease [23].

At the moment we are entering the era of regimens combining immune checkpoint inhibitors with TKIs, it is crucial to realize that combining these drugs results in increased prevalence of treatment related adverse events. In the open-label, dose-escalation, phase I CheckMate 016 trial combining nivolumab plus sunitinib or pazopanib, hepatic impairment was registered in 45.5% (grade 3 and 4 in 24.2%) patients in nivolumab + sunitinib arm and 35% (grade 3 and 4 in 20%) in nivolumab + pazopanib arm [24]. Due to extensive off-target activity and associated toxicity of MTKIs, other TKIs such as axitinib or lenvatinib have been later selected for combination regimens with immune checkpoint inhibitors in mRCC [25].

Sunitinib is significantly metabolized by the cytochrome P450 3A4 (CYP3A4). Strong CYP3A4 inhibitors (e.g. clarithromycin) or inducers are able to cause a clinically relevant modification in plasma concentrations of sunitinib and these interactions may potentially lead to increased adverse effects and toxicity or treatment failure [26].

Herein we report two exceptional cases of severe liver injury induced by TKIs. The first case report documents a rare case of severe pazopanib-induced liver injury with subsequent manifestation of AML two years later. Coincidence of grade 4 liver injury and subsequent diagnosis of AML has not been reported yet. The second case report describes an uncommon case of lethal acute liver failure possibly due to interaction between sunitinib and clarithromycin. To the best of our knowledge, lethal acute liver failure possibly induced by clarithromycin has not yet been published in the literature.

## Case presentation

### Case 1

A 70-year old male patient presented in July 2012 with multiple metastases affecting both lungs. The patient had a history of right-sided kidney tumor that was radically removed in March 2007. Histological examination of the primary tumor revealed poorly differentiated pT3 clear-cell RCC. Laboratory examinations at the time of metastatic disease presentation in July 2012 showed no abnormality. The medical history was otherwise unremarkable with no concurrent medication. Because of the toxicity and non-curative nature of systemic therapy, taking into consideration so far indolent behavior of the disease and patient preference, the strategy of active surveillance was selected initially.

**Fig. 1:**
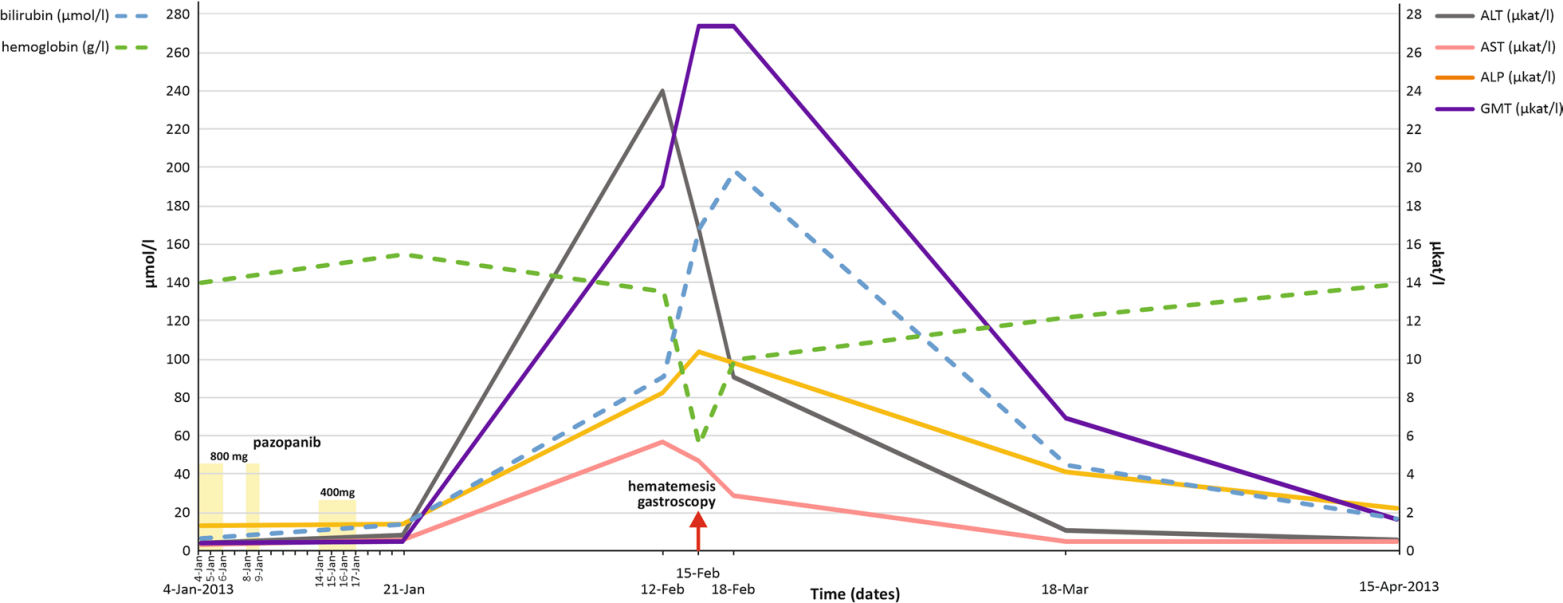
Graph showing the changes in liver function tests for case 1, with a summary of systemic therapy given over time

A computed tomography (CT) examination 6 months later showed new lung lesions and increase in size of the prior pulmonary metastases. At this point, after the consultation with the patient it was decided to initiate systemic treatment. The patient had a good prognostic score according to the International Metastatic Database Consortium (IMDC). The treatment with pazopanib was initiated in January 2013 at the standard dose of 800 mg OD (Fig. 1). The laboratory parameters of liver function were normal at baseline. Within the first three days of pazopanib administration the patient reported asthenia, hypertension and significant nausea. The treatment was immediately interrupted. After hypertension and nausea were controlled by medications including oral metoclopramide and perindopril, the administration of pazopanib was continued at the same dose level. Two days later, the therapy was again complicated by grade 3 nausea according to Common Terminology Criteria for Adverse Events, version 4.03 (CTCAE 4.03). Pazopanib was interrupted until the symptoms resolved. The treatment was restarted at dose of 400 mg OD, but continued only for 4 days and then permanently stopped because of symptom recurrence. The patient used pazopanib for a total duration of 9 days. After treatment termination, the patient felt relieved, but 3 weeks after the treatment termination (35 days from pazopanib initiation) developed nausea, vomiting and grade 4 ALT elevation, hyperbilirubinaemia (˃ 20xULN), and hypereosinophilia (Fig. 1). Other laboratory tests were within the normal range. The patient had no history of liver disease or iron overload. Alternate causes of acute liver enzyme elevation, including cytomegalovirus infection (CMV), hepatitis A, B, C, E or autoimmune hepatitis were excluded. The abdominal ultrasound showed normal findings. The patient received hepatoprotective medications including silymarin and essential phospholipids, but was advised that the efficacy was controversial and disputed. The patient complained of hematemesis and melena 10 days later. Endoscopy identified reflux esophagitis grade D according to the Los Angeles classification and two peptic ulcers (Forrest IIb and Forrest IIc) in the stomach antrum and the angular notch (Fig. 1). The bleeding was stopped by an adrenalin injection and the patient received proton pump inhibitors. Hemoglobin concentration was 56 g/l and the patient received transfusions (Fig. 1). Liver biochemistry abnormalities gradually recovered to normal and all clinical symptoms disappeared. The RCC course was not followed by any radiographic examinations as the patient refused further treatment. Subsequently, on October 21, 2015 the patient was diagnosed with AML. Due to poor prognostic profile of leukaemia, patient age and comorbidity, no curative treatment was administered for leukaemia and only symptomatic treatment was recommended. The patient died of leukemia on June 17, 2016.

### Case 2

**Fig. 2:**
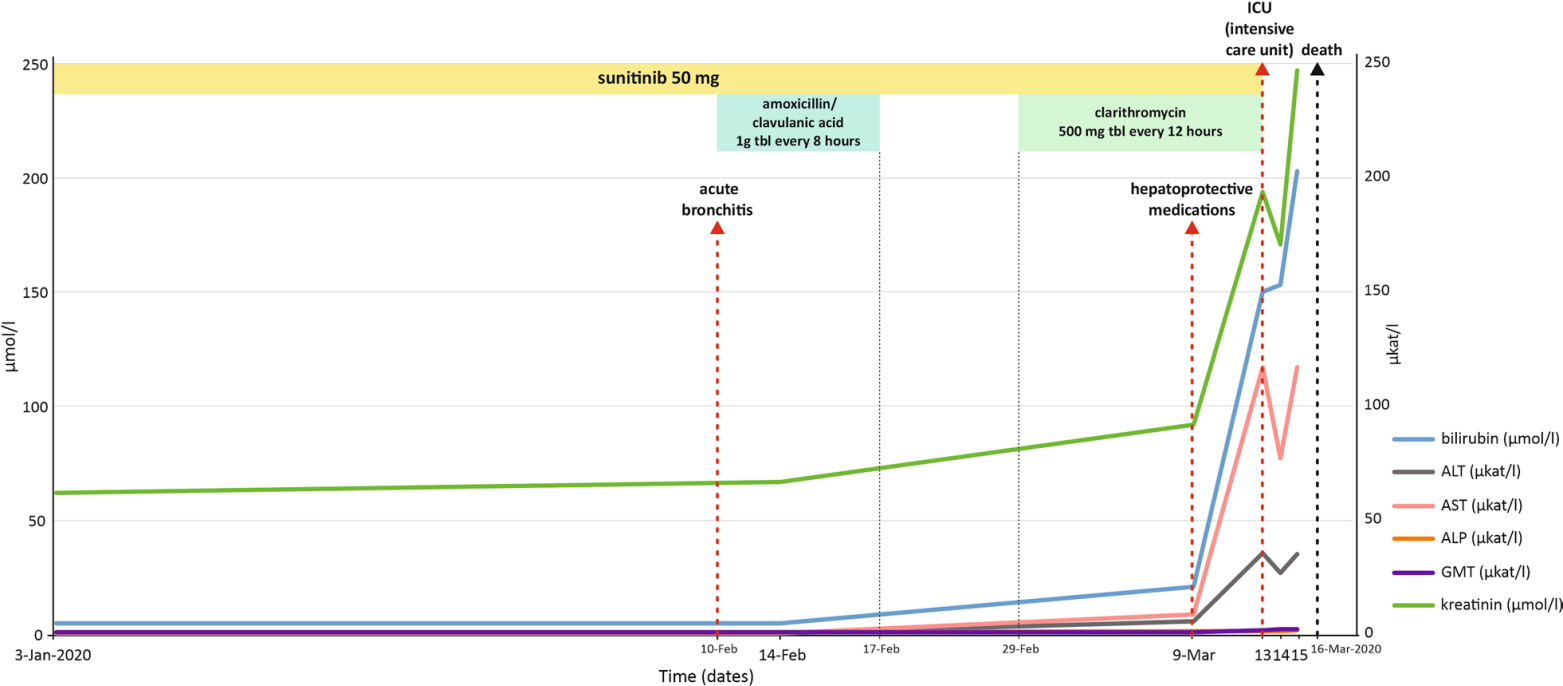
Graph showing the changes in liver function tests for case 2, with a summary of systemic therapy given over time

A 44-year old female patient presented in December 2014 with abdominal pain. CT showed a tumor of the right kidney, 7 cm in size. The medical history was otherwise unremarkable with no concurrent medication. The tumor was radically removed in January 2015. Histological examination revealed moderately differentiated pT1a clear-cell RCC. Two solitary lung metastases were diagnosed in October 2016 and resected with curative intent in November 2016. Histological examination confirmed well differentiated clear cell carcinoma. Positron emission tomography-computed tomography (PET/CT) revealed new multiple lung lesions in September 2017. Laboratory examinations showed no abnormality, and the patient had a good prognosis score according to IMDC. The treatment with sunitinib was initiated in September 2017 at the standard dose of 50 mg OD for 4 weeks followed by 2 weeks off treatment (schedule 4/2). The laboratory parameters of liver function were normal at baseline. During the first cycle of therapy the patient was suffering from grade 2 hypertension, grade 2 dyspepsia and grade 2 hypothyreodism. The patient continued with the therapy and the tolerance of the therapy gradually improved while hypertension, dyspepsia and hypothyreodism were controlled by medications including oral metoclopramide, perindopril with amlodipine and levothyroxine. Apart from mild complications such as grade 1–2 diarrhea for a short period of time during the course therapy and grade 1 hand-foot syndrome, the patient tolerated sunitinib therapy at full dose quite well achieving partial response on CT according to Response Evaluation Criteria in Solid Tumors (RECIST). In February 2020, the patient had acute bronchitis that was initially treated with amoxicillin/clavulanic acid and ambroxol with no improvement. Amoxicillin/clavulanic acid was replaced by clarithromycin by a physician in the emergency unit which the patient visited for worsening of the cough (Fig. 2). Ten days later, the patient visited the emergency unit again for abdominal pain and asthenia. The lab test showed grade 4 ALT and aspartate aminotransferase (AST) elevation, while other laboratory tests were within normal range (Fig. 2). The patient received hepatoprotective medications including silymarin and essential phospholipids (Fig. 2) and was sent home to have the lab tests checked by the general practitioner. Four days later, the patients was admitted to the hospital for acute liver failure with hyperbilirubinaemia (˃ 7xULN), grade 4 ALT (˃ 40xULN) and AST (˃ 180xULN) elevation, coagulopathy and grade 4 thrombocytopenia. The patient was transferred to intensive care unit (Fig. 2). Alternate causes of acute liver injury including CMV, hepatitis A, B, C, E and autoimmune hepatitis were excluded. All medications including sunitinib were interrupted upon the admission to the hospital. The patient continued with hepatoprotective medications. Liver transplantation was considered contraindicated because of active malignancy. The overall clinical status along with LTs abnormalities and other parameters gradually worsened and the patient died three days later on March 16, 2020 with clinical and laboratory signs of liver necrosis (Fig. 2). The autopsy was not performed.

## Discussion and conclusions

Liver injury is a feared adverse effect of MTKI. The incidence of liver injury varies with individual agents, but there may be an increased risk in combination, in particular with immune checkpoint inhibitors [5].

Currently, the underlying mechanisms of TKI-induced liver injury are only partially clarified. A class effect based on inhibition of a specific tyrosine kinase is unlikely because pharmacologically and structurally diverse TKIs are known to be hepatotoxic. Although these can target the same tyrosine kinase or belong to the same chemical class, available evidence suggests a lack of cross-reactivity between TKIs. In case of acute or chronic hepatocyte injury studies have shown that injury caused by TKIs was mainly associated with hepatocellular damage, rather than cholestasis [27, 28].

A given drug may cause toxic effects by several mechanisms including inhibition of glycolysis, impaired oxygen consumption, mitochondrial dysfunction and induction of apoptosis in hepatocytes [29]. Mitochondrial toxicity of sunitinib is connected with reduced mitochondrial membrane potential and increased mitochondrial reactive oxygen species production leading to reduced cellular glutathione (GSH) pool which can induce mitochondrial oxidative stress and apoptosis [30]. Emerging reactive intermediate metabolites can also contribute to cellular TKIs toxicity, meanwhile causing direct toxicity through binding to neighboring proteins and macromolecules. Consequently, indirect toxicity may be secondary as immune reactions recognize structurally altered proteins as a foreign macromolecule and serve as haptens [31]. DILI is traditionally classified as intrinsic (or direct) vs. idiosyncratic. Intrinsic DILI is typically dose-related, predictable, affects a large proportion of exposed individuals, since the drug is toxic at a given threshold level. Onset of the toxicity exhibits within a short time period (hours to days). On the other hand, idiosyncratic DILI is usually not dose-related, unpredictable, affects only a small proportion of individuals exposed to the drug and occurs with a variable latency to onset of days to weeks [32]. MTKIs can cause both type of liver injury, as shown in the present case reports.

Causality scores such as RUCAM are intended to confirm or exclude the suspicion of DILI. RUCAM is a well-established diagnostic algorithm using a scale to assess causality in patients with suspected DILI. RUCAM has become the most widely used method to advocate DILI diagnosis in different settings owing to clear definition and classification of each case of liver injury. Moreover, it consists of precise criteria as well as a scoring system to validate the original method [33].

RUCAM was first published in 1993. The worldwide experience with the original RUCAM was the principal factor facilitating modification of the original RUCAM, which resulted in the publication of current version, the updated RUCAM in 2016 [34]. Based on updated RUCAM, pazopanib may be considered a probable cause of DILI in the first case report presented here. RUCAM was applied retrospectively in this case. Subscale for the cholestatic or mixed type of injury of updated RUCAM was used because ratio R, calculated as the ALT/ alkaline phosphatase (ALP) activity measured at the time liver injury was suspected, was 3 (mixed liver injury). Total score for this case was 8 indicating causality grading as probable. Application of RUCAM to the second case presented here is complicated by the interaction between sunitinib and clarithromycin. RUCAM was also applied retrospectively. Subscale for the hepatocellular injury of updated RUCAM was used because ratio R, calculated as the ALT/ ALP activity measured at the time liver injury was suspected, was 23. The total score in this case for sunitinib was 1 providing causality grading of unlikely. This is consistent with our opinion that the culprit of the toxicity was not sunitinib alone, but rather the interaction of sunitinib with clarithromycin. In the second case, we also applied RUCAM for amoxicillin/clavulanic acid, which preceded clarithromycin in treatment of acute bronchitis, and the score for amoxicillin/clavulanic acid was 2, i.e. unlikely causality. Identifying culprit drugs and individuals at risk for DILI remains challenging. Not only genetic factors predisposing individuals at risk, but also the role of the physicochemical and toxicological properties and the drug interactions with the host and environmental factors need to be considered. Mechanisms involved in DILI can be multifactorial [35].

At present, intervention strategies for liver toxicity of TKIs consist generally of dose adjustment and discontinuation, combined with conventional treatment strategies. To ascertain the risk of serious drug-induced liver injury and the need to discontinue the suspect drug, Hy’s law is widely used [36]. According to the Hy’s law isolated hepatocellular injury (i.e. without a significant obstructive component) sufficient to cause hyperbilirubinemia is an ominous sign of the potential to cause serious liver injury. For patients to meet Hy’s law criteria means having ALT or AST levels ≥ 3 times ULN along with total bilirubin ≥ 2 times ULN without initial findings of cholestasis (i.e. absence of elevation of ALP > 2 ULN) and excluding any other causes to be involved in the liver injury. When the above criteria for acute liver failure have been met, Hy’s law predicts that this liver injury leads to death or liver transplantation in > 10% of cases [32, 36, 37].

Biomarkers play a crucial role in the management of cancer patients and are urgently needed for both risk prediction and monitoring of liver injury. Several genetic risk factors for idiosyncratic adverse drug reaction have been identified. Preceding pharmacogenetic studies have reported association between Gilbert syndrome uridine diphosphate glucuronosyltransferase 1A1 (UGT1A1) variants and hyperbilirubinemia in patiens treated with pazopanib and sunitinib [38, 39]. The UGT1A1 enzyme is inhibited by pazopanib, and patients carrying the UGT1A1∗28 allele have been identified to be at risk of hyperbilirubinemia [40]. Xu et al. reported on a potential association between the presence of a mutation of the hemochromatosis gene (HFE) on chromosome 6 and human leukocyte antigen B∗57:01 (HLA-B∗57:01) carrier status, and ALT elevation in pazopanib-treated patients [41]. Genetic characterization of treatment-related LTs elevations may elucidate the underlying mechanism and the nature as well as the risk of DILI. This may allow tailored management in individual patients, including continued treatment for many patients, without increasing the risk of serious DILI [42].

Pazopanib is predominantly metabolized in the liver by CYP3A4 and 1A2 pathway and liver injury may be related to production of its toxic metabolites. In vitro and in vivo test identified over 20 pazopanib metabolites, including several cysteine adducts and aldehyde derivatives which may cause oxidative stress and can be potentially responsible for liver injury [43, 44]. However, there is some evidence indicating immune system involvement that is not linked to pazopanib pharmacokinetics and dose [41].

According to the manufacturer guidelines, patients with elevated transaminases of ˃8xULN should interrupt pazopanib until LTs return to ≤ 3xULN. Thereafter, pazopanib can be reintroduced at a reduced daily dose of 600 mg. In case of LTs elevation recurrence, pazopanib should be permanently discontinued. If autoimmune inflammation is considered as the potential cause of liver injury, corticosteroids could be the treatment of choice as reported by Vlenterie et al. [45]. If there is no therapeutic option, treatment with an alternative TKI such as sunitinib with a different toxicity profile may be applied under close monitoring of LTs in patients with pazopanib-induced liver injury [46].

Suttle et al. published a retrospective analysis of 177 mRCC patients showing an increased tumour shrinkage and longer PFS in patients with plasma trough levels (C_min_) ≥ 20.5 mg/L compared with patients with a C_min_ below this threshold [47]. Median PFS in patients with higher pazopanib Cmin was reported to be 50.2 weeks versus 19.6 weeks in patients with lower Cmin, while an association was also observed in median tumor shrinkage 37.9% in the high versus 6.9% in the low exposure group. These data indicate a strong association between the efficacy of pazopanib and pharmacokinetic exposure as previously reported in a preclinical study [48]. Currently, 20% patients taking the approved daily dose of 800 mg do not reach the threshold and may be at risk of ineffective treatment.

Significant interindividual variability in plasma exposure has been observed in pharmacokinetic studies in addition to a potential influence of large variability in exposure resulting from concomitant medication, food intake and patient compliance [49, 50]. High risk of suboptimal treatment outcomes in a subset of patients with a low C_min_ while taking the currently approved fixed dose of pazopanib should be considered. Meanwhile, patients with a significant toxicity requiring dose reduction could maintain adequate exposure in spite of the reduction of pazopanib dose. Promising data from prospective trials have displayed some benefit of individualized strategy of pazopanib dosing, but further investigations are needed [51–53]. Similarly to imatinib, a time-dependent decrease in exposure was observed in patients treated continuously with pazopanib possibly as a result of upregulated drug transporters or CYP3A4 [51, 54, 55].

Sunitinib is predominantly metabolized by CYP3A4 and CYP1A2, and factors that alter CYP3A4 and CYP1A2 activity may affect patient risk for sunitinib toxicity. Formation of reactive metabolites from sunitinib based on detection of GSH conjugates was described in vitro, however, until recently the structures of the reactive metabolites were not characterized [56].

Amaya et al. reported in vitro identification of CYP3A4 enzymes involved in sunitinib metabolic pathways. Products of sunitinib biotransformation identified are N-desethylsunitinib (M1), monooxygenated metabolites (M2), defluorinated sunitinib (M3), and glucuronide conjugates (M4). The defluorinated metabolite M3 contains a *para*-hydroxyaniline moiety, which can be further oxidized to an electrophilic reactive and potentially toxic quinoneimine (M5). CY3A4 is primarily responsible for formation of M1, the major active metabolite of sunitinib. CYP1A2 and CYP3A4 are both involved in formation of M5, the quinoneimine metabolite. CYP1A2 has greater efficiency for formation of quinoneimine compared to CYP3A4. Formation of quinoneimine is higher in human liver microsomes with high CYP1A2 activity compared to human liver microsomes with low CYP1A2 activity. Smoking may also increase the generation of sunitinib reactive metabolites since smoking is known to induce CYP1A enzymes. Moreover, a chemically reactive moiety is incorporated in sunitinib and its main active metabolite M1, which can undergo addition with cellular thiols, such as cysteine residues of proteins and GSH. Thus, conjugates with GSH can be formed directly from the parent drug and M1 metabolite and both may contribute to GSH depletion, increasing toxicity of sunitinib [57]. Zhao et al. recently reported that four reactive metabolites along with impaired clearance of sunitinib in liver played a dominant role in sunitinib‐induced liver injury [58]. Paludetto et al. published their observation from human plasma samples obtained during drug monitoring where sunitinib and pazopanib aldehyde reactive metabolites with high electrophility and reactivity toward proteins were identified [44].

Clarithromycin, a commonly used macrolide antibiotic, is a potent CYP3A4 inhibitor. Irreversible inhibition of CYP3A4 caused by clarithromycin leads to its inactivation and a new CYP3A4 protein has to be synthesized to replace it. Contrary to reversible inhibition of CYP3A4, mechanism-based inhibition of CYP3A4 more frequently causes drug-drug interactions [59]. Clarithromycin, just as other macrolide antibiotics, has been related to a low frequency of acute, transient and usually asymptomatic increase in serum aminotransferase levels in 1% to 2% of patients treated for short periods and a somewhat higher number of patients after long-term clarithromycin taking. Asymptomatic increase in serum liver enzymes can be commonly noticed among elderly patients, especially given higher doses of clarithromycin. Clarithromycin has also been linked to acute, clinically apparent liver injury with jaundice, which is observed to occur in 3.8 per 100,000 prescriptions. Clarithromycin induced liver injury is typically represented by cholestatic hepatitis, nevertheless uncommon cases with hepatocellular injury and sudden onset have also been mentioned in the literature [60].

The published literature information on sunitinib drug interactions with other drugs is currently scarce, and based mostly on the results of in vitro assays, animal studies or pharmacokinetic measurements after the administration of a single dose in healthy volunteers, and it is rather difficult to apply such information to clinical practice. Thus, a careful medical evaluation of each individual patient is essential [61]. Szalek et al. reported no effect of a single oral dose of clarithromycin or azithromycin on the pharmacokinetics of sunitinib in rabbits [62]. A retrospective study evaluating the association between clinically relevant toxicities of pazopanib and sunitinib and the use of weak CYP3A4 and P-gp inhibitors in 76 patients found a significant correlation between the use of inhibitors and the dose reduction or withdrawal of TKIs [63].

European Summary of Product Characteristics of Sutent in healthy volunteers states that concomitant administration of a single dose of sunitinib with ketoconazole, the potent CYP3A4 inhibitor, led to an increase of the combined (sunitinib + primary metabolite) maximum concentration (Cmax) and area under the curve (AUC) values by 49% and 51%, respectively. Therefore, it is recommended that co-administration of sunitinib with potent CYP3A4 inhibitors should be avoided if possible due to risk of an increase in plasma concentration of sunitinib. In case the co-administration cannot be eluded the dose of sunitinib should be considered to be lowered to a minimum of 37.5 mg daily for GIST and mRCC or 25 mg daily for pNET, provided careful monitoring of adverse events.

It may be assumed from the pharmacokinetic properties of sunitinib and clarithromycin that the co-administration of clarithromycin has a potential to result in an accumulation of sunitinib and its reactive metabolites, which may lead to liver injury. It can be considered that the inhibition of CYP3A4 may enhance biotransformation of sunitinib via CYP1A2, which has greater efficacy for formation of toxic quinoneimine. It is questionable whether hepatotoxic effect of clarithromycin alone might have been a contributory factor in the second case reported here, but sunitinib as a causative agent seems plausible due to primary hepatocellular type of liver injury and properties of the drugs.

The general management of DILI consists of the discontinuation of the offending drug in combination with supportive treatment. Therapeutic re‐challenge with the suspicious drug is generally not advisable, but may be attempted in certain instances after a thorough consideration of the risks and potential benefits. Specific therapies available for DILI are limited to carnitine for valproic acid, N‐acetylcysteine (NAC) for acetaminophen overdose and cholestyramine for leflunomide. If liver injury is immune-mediated, then corticosteroids may be useful for the management of the toxicity [32]. NAC is an antioxidant agent that replenishes mitochondrial and cytosolic glutathione stores. The benefit of NAC application for non-paracetamol drug-induced liver failure has been discussed over the years and recommendations differ. NAC should be considered for patients with early stage of non-acetaminophen drug-induced acute liver failure and is commonly used because of potential benefit and an acceptable safety profile [32, 64–66]. The contribution of NAC was established in a randomized placebo-controlled trial demonstrating the transplant-free survival of individuals with non-acetaminophen induced acute liver failure and grade 1–2 coma who received NAC was significantly higher than those who did not receive NAC [67]. However, another trial showed no efficacy [68]. Kortsalioudaki et al. report in a retrospective study that NAC administration was associated with an improved outcome including shorter length of hospital stay, higher incidence of native liver recovery without transplantation, and better survival after transplantation [69]. Meta-analysis indicated safety and limited benefit regarding prolongation of patient survival with native liver without transplantation and survival after transplantation however, with no improvement of overall survival [70] NAC significantly reduces the level of reactive oxygen species produced by sunitinib and crizotinib and reduces sunitinib- and crizotinib-induced mitochondrial apoptosis and cellular damage. The use of NAC has been reported in several case reports of fulminant acute liver failure associated with TKIs [17, 71–73].

Subsequent metachronous second primary malignancies are usually not expected in patients with mRCC, and we may only speculate whether there was any association between pazopanib treatment and AML manifestation. Such associations are well documented for cytotoxic agents [74]. In contrast to cytotoxic drugs, targeted agents have been used on a wider scale only during the last ten years or so, mostly in patients with advanced disease and limited survival expectations, and these stochastic events may just begin to emerge.

In conclusion, the first case report describes severe liver injury induced by pazopanib in a mRCC patient who died of AML three years later. This case illustrates an urgent need for biomarkers to identify patients at high risk of developing significant liver injury aiming at optimal treatment selection with a regard to maximal efficacy and minimal adverse effects.

The second case report describes an uncommon case of fatal acute liver failure possibly due to pharmacokinetic interaction between sunitinib and clarithromycin. Drug-drug interactions comprise an important issue in medical oncology. Over the past years, there has been a shift in cancer treatment from the traditional time-limited administration of nonspecific cytotoxic agents to the prolonged treatment with numerous new targeted drugs. Targeted agents, in particular TKIs, are typically administered continuously or with relatively brief interruptions orally and are metabolized by CYP3A4, leading to a high risk of drug-drug interactions. It is essential to monitor patients receiving TKIs for potential drug-drug interactions to avoid the risk of toxicity or loss of efficacy. In the era of precision medicine, the time has come to administer not only the right therapeutic agent to the right person, but also with a regard to dosing of drugs, in particular TKIs, the approach should be personalized [75, 76].

## Data Availability

The authors declare that all data concerning this case report are provided within the manuscript.

## Abbreviations

MTKI: Multityrosine kinase inhibitor
RCC: Renal cell carcinoma
TKI: Tyrosine kinase inhibitor
DILI: Drug-induced liver injury
RUCAM: Roussel Uclaf causality assessment method
mRCC: Metastatic renal cell carcinoma
ALT: Alanine aminotransferase
AML: Acute myeloid leukemia
VEGFR: Vascular endothelial growth factor receptors
SCF: Stem cell factor receptor
PDGFR: Platelet-derived growth factor receptors
PFS: Progression-free survival
FLT3: Fms-like tyrosine kinase-3
CSF-1R: Colony stimulating factor receptor
RET: The glial cell-line derived neurotrophic factor receptor
GIST: Gastrointestinal stromal tumor
pNET: Pancreatic neuroendocrine tumor
OD: Once daily
CYP3A4: Cytochrome P450 3A4
CT: Computed tomography
IMDC: International Metastatic Database Consortium
CTCAE: Common Terminology Criteria for Adverse Events
ULN: Upper limit of normal
CMV: Cytomegalovirus infection
PET/CT: Positron emission tomography-computed tomography
RECIST: Response Evaluation Criteria in Solid Tumors
AST: Aspartate aminotransferase
GSH: Glutathione
ALP: Alkaline phosphatase
UGT1A1: Uridine diphosphate glucuronosyltransferase 1A1
HFE: Hemochromatosis gene
HLA-B∗57:01: Human leukocyte antigen B∗57:01
Cmin: Plasma trough levels
Cmax: Maximum concentration
AUC: Area under the curve
NAC: N‐acetylcysteine
